# Plasmonic Coupling for High‐Sensitivity Detection of Low Molecular Weight Molecules

**DOI:** 10.1002/smsc.202400382

**Published:** 2024-10-09

**Authors:** Alexa Guglielmelli, Rossella Zaffino, Giovanna Palermo, Liliana Valente, Dante Maria Aceti, Loredana Ricciardi, Arántzazu González‐Campo, Raphael Pfattner, Núria Aliaga‐Alcalde, Giuseppe Strangi

**Affiliations:** ^1^ Department of Physics NLHT‐Lab University of Calabria and CNR‐NANOTEC, Institute of Nanotechnology 87036 Rende Italy; ^2^ Institut de Ciència de Materials de Barcelona (ICMAB‐CSIC) Campus Universitari 08193 Bellaterra Spain; ^3^ CNR‐NANOTEC, Institute of Nanotechnology 87036 Rende Italy; ^4^ ICREA (Institució Catalana de Recerca i Estudis Avançats) Passeig Lluïs Companys 23 08010 Barcelona Spain; ^5^ Department of Physics Case Western Reserve University 2076 Adelbert Rd. Cleveland OH 44106 USA

**Keywords:** microfluidic device, plasmonic coupling, plasmonic modes, sensing, small molecules detection

## Abstract

This article presents a novel plasmonic sensing platform designed for the detection of low molecular weight molecules, offering significant advancements in diagnostic applications. The platform features a periodic array of gold nanodisks on a 20 nm thin silica layer, supported by a 100 nm thick gold substrate. By leveraging the coupling between localized and propagating surface plasmon resonances, this design significantly enhances the sensitivity and specificity of molecular detection. Finite element method simulations are conducted to characterize the optical properties and reflectance response of the nanodisks array in the visible to near‐infrared range. Ellipsometric analysis is performed to measure the reflectance of the sample at various angles. Additionally, scanning near‐field optical microscopy in reflectance mode validates the design by revealing well‐defined plasmonic hot spots and interference patterns consistent with the simulated results. The findings demonstrate the platform's effectiveness in amplifying optical signals, achieving a limit of detection of 50 μM for molecules with a molecular weight of less than 1 KDa. This high sensitivity and specificity highlight the potential of the proposed plasmonic platform to advance the development of highly sensitive sensors for low molecular weight molecules, making it a valuable tool for diagnostics and precise molecular detection.

## Graphical abstract

This work presents a novel plasmonic sensing platform designed for the detection of low molecular weight molecules, offering significant advancements in diagnostic applications. The platform features a periodic array of gold nanodisks on a SiO_2_/Au bilayer. By exploiting the coupling between localized and propagating surface plasmon resonances, this design significantly enhances the sensitivity and specificity of molecular detection.

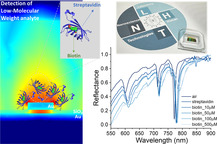

## 1 Introduction

The detection of molecules with low molecular weight (<1 KDa) is a critical challenge in various fields, including environmental monitoring,^[^ ^1^, ^2^, ^3^, ^4^, ^5^ ^]^ medical diagnostics,^[^ ^6^, ^7^, ^8^, ^9^, ^10^ ^]^ and biochemical research.^[^ ^11^, ^12^, ^13^, ^14^ ^]^ Traditional sensing methods often struggle with sensitivity and specificity at such low scales, necessitating the development of advanced techniques capable of detecting these molecules with high accuracy and reliability. In this context, plasmonic sensing platforms have emerged as powerful tools due to their ability to enhance electromagnetic fields at the nanoscale, thereby increasing the interaction between light and analytes.^[^ ^15^, ^16^, ^17^, ^18^, ^19^, ^20^ ^]^ Plasmonic sensors exploit surface plasmon resonances (SPRs), which are collective oscillations of free electrons at the interface between a metal and a dielectric.^[^ ^15^, ^21^, ^22^ ^]^ These resonances can be categorized into two main types: localized surface plasmon resonances (LSPRs) and propagating surface plasmon polaritons (SPPs).^[^ ^11^, ^22^, ^23^, ^24^ ^]^ LSPRs are confined to metallic nanoparticles and exhibit strong field enhancement in the proximity of the surface, making them highly sensitive to changes in the local refractive index (RI). However, their detection range is limited to the immediate vicinity of the nanoparticles.^[^ ^25^ ^]^ In contrast, SPPs are surface waves that propagate along a metal–dielectric interface, allowing for the detection of changes over larger areas but generally offering lower field enhancement compared to LSPRs.^[^ ^26^ ^]^

In the field of biosensing, plasmonic biosensors are of particular interest, especially SPPs and LSPR sensors. Experimental SPP sensors have exhibited bulk sensitivity around 5.9 × 10^4^ nm RIU^−1^(RI unit), with theoretical predictions reaching up to 2 × 10^5^ nm RIU^−1^.^[^ ^27^, ^28^, ^29^, ^30^ ^]^ LSPR sensors, while smaller and suitable for point‐of‐care testing, typically achieve sensitivities ranging from 100 to 658 nm RIU^−1^ for bulk RI sensing.^[^ ^28^, ^31^, ^32^, ^33^, ^34^ ^]^ Dielectric sensors, in contrast, generally reach a typical bulk sensitivity up to 300 nm RIU^−1^.^[^ ^35^, ^36^ ^]^ An all‐dielectric crescent metasurface sensor reached a sensitivity up to 326 nm RIU^−1^.^[^ ^37^ ^]^ This sensitivity, achieved through higher‐order resonances, surpasses the previously reported quasi‐bound states in the continuum‐driven sensing of 263 nm RIU^−1^ in the visible region.^[^ ^38^ ^]^ However, hybrid metal–dielectric nanostructures have gained prominence for their ability to combine the strong field enhancement of plasmonic metals with the low‐loss radiation channels of dielectric resonators. For instance, nanoantennas combining Si cylinders with Al disks separated by SiO_2_ spacers have been experimentally modified to increase the sensitivity to 245 nm RIU^−1^.^[^ ^39^ ^]^ Additionally, plasmonic 3D metasurface structures have been numerically tested, reaching a sensitivity of 761 nm RIU^−1^. However, fabricating such 3D metasurface structures is not a trivial task.^[^ ^40^ ^]^ While, techniques like photolithography, electron beam lithography, and etching are suitable for making 2D metasurfaces. 3D metasurfaces require even more advanced fabrication like laser 3D nanoprinting to enable arbitrary designs.^[^ ^41^ ^]^

In this article, we present a sensing platform that synergistically integrates LSPRs and SPPs to overcome the limitations of each individual approach. By combining the high sensitivity of localized modes with the extensive detection range of propagating modes, our hybrid system achieves superior sensitivity and specificity in detecting low molecular weight molecules, addressing a significant need in analytical chemistry and biomedicine. We demonstrate the effectiveness of our platform through both numerical simulations and experimental validations. Sensitivity is calculated from a calibration sensing test using glycerol, while specificity is verified through a second test using the streptavidin‐biotin model system to detect small molecules. Our comprehensive analysis highlights the pivotal role of the coupling between LSPRs and SPPs, which not only enhances detection limits but also significantly improves the robustness and reproducibility of the sensor. By elucidating this hybrid plasmonic sensing approach, we aim to advance sensitive and reliable detection technologies for low molecular weight molecules. This advancement has the potential to facilitate breakthroughs in early disease diagnosis and the development of innovative nanomaterials for various applications in biomedicine and environmental monitoring.

## 2 Results and Discussion

**Figure 1:**
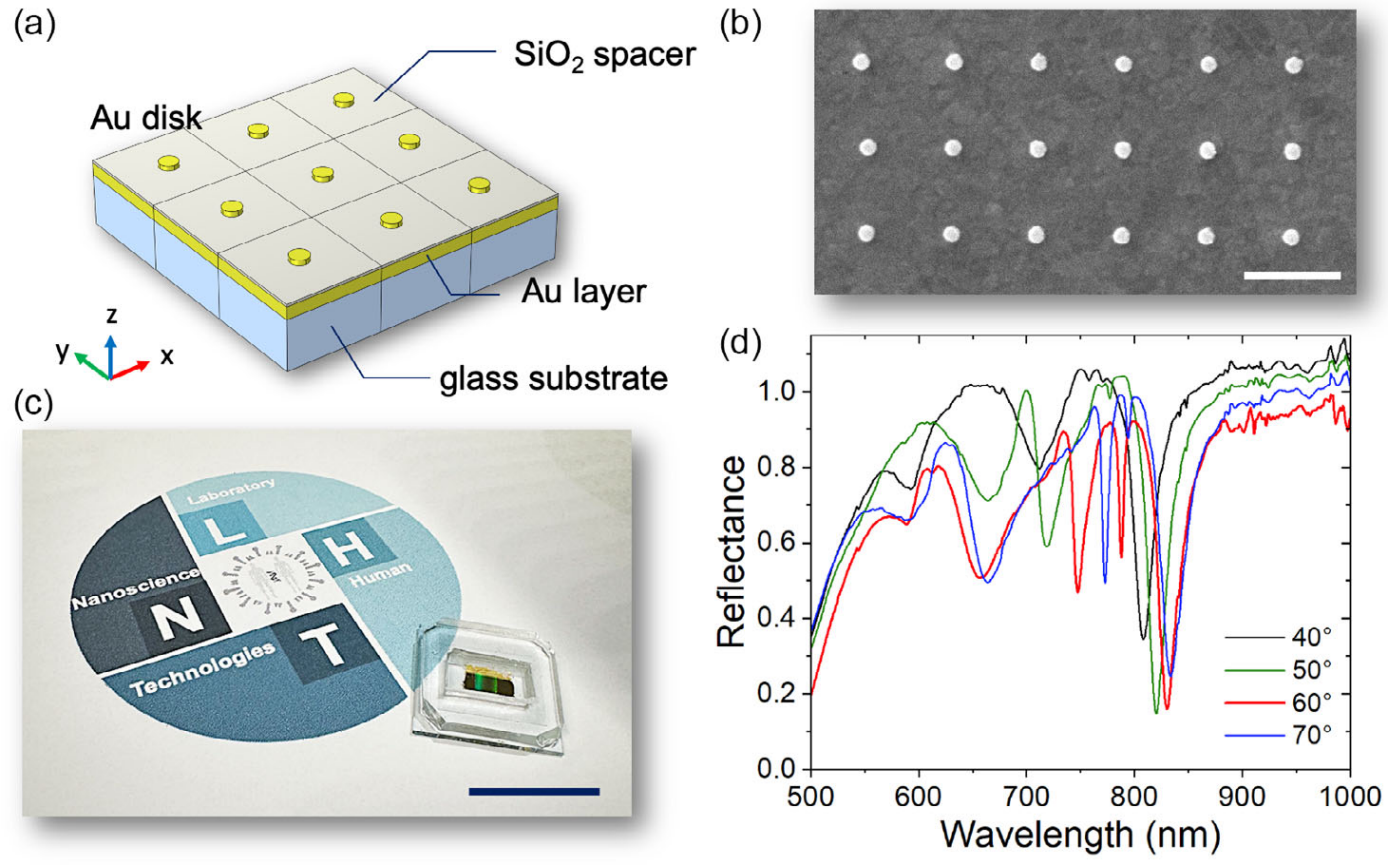
Au nanodisk‐based metasurface. a) Schematic illustration of the Au nanodisk array, along with b) the corresponding SEM image of the fabricated sample (scale bar, 1 μm). c) Representative photo of the sensor device, integrated with a microfluidic chamber (scale bar, 2 cm). d) Reflectance spectra of the metasurface recorded at various angles of incidence.

As reported in the schematization of **Figure** **1**a, the sensing platform is composed of a squared array of Au nanodisks with a diameter of 150 nm and an height of 40 nm on an SiO_2_ layer (20 nm); supported on a 100 nm Au layer. The periodicity in the *x*‐*y* plane of the array is around 780 nm, as we can see in the scanning electron microscopy (SEM) image (Figure 1b). The sensor area is integrated in a microfluidic chamber (Figure 1c) to allow testing the platform sensitivity performance through the use of small volumes of fluids. The reflectance spectra of the plasmonic metasurface, for p‐polarized light, were measured at various incidence angles ranging from 40° to 70°, as illustrated in Figure 1d. The results reveal several distinct reflectance minima within the 500–1000 nm wavelength range. All modes depend significantly on the change in the angle of incidence, showing red‐shifting with increasing angle. As can be seen from the curves in Figure 1d, the modes characterized by a higher Q‐factor occur for the incidence angle of 60°. For this reason, in the rest of the analysis, we will consider this as the angle of incidence.

### 2.1 Finite Element Method (FEM) Analysis

To investigate the optical properties of the structure, we employ FEM to calculate the near‐field intensity distribution. The modeled geometry is illustrated in the Supporting Information (SI). A unit cell composed of multiple layers is constructed using COMSOL Multiphysics, specifically utilizing the electromagnetic waves and frequency domain module to compute both the reflectance spectrum, and the near‐field response of the structure. We simulate light incidence on the structure using a plane wave source, configured for both normal and angled incidence. The incident wave is characterized by s‐polarization and p‐polarization. To model the infinite periodic array, we apply periodic boundary conditions (Floquet periodicity) in the *x*‐ and *y*‐directions, while perfectly matched layer boundary conditions are implemented in the *z*‐direction to effectively absorb outgoing waves. The RI of the glass substrate is set to 1.517.

**Figure 2:**
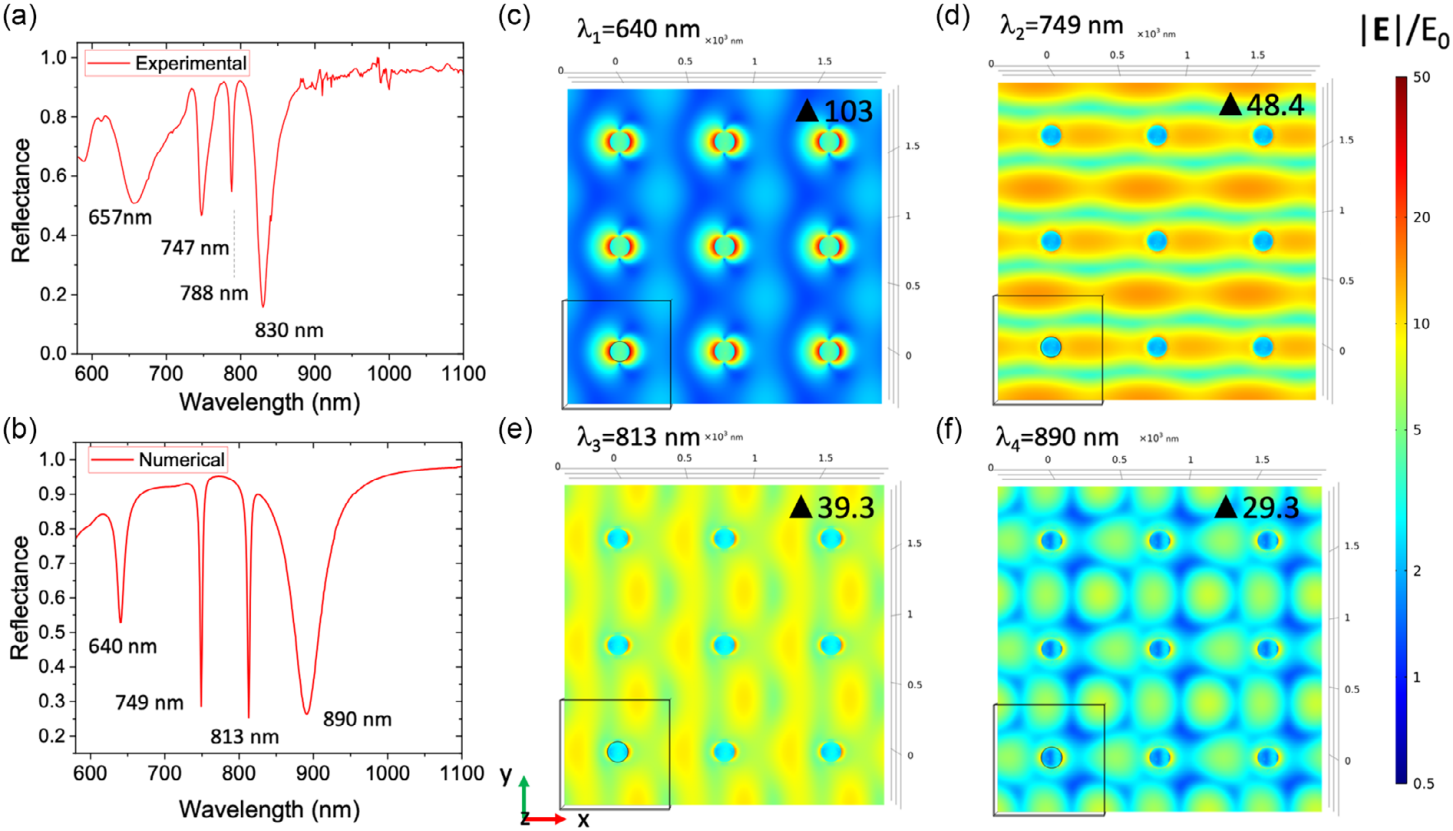
Plasmonic response of the composite structure. a) Experimental and b) simulated reflectance spectra with air as surrounding medium at 60° angle of incidence. FEM numerical results of the spatial distribution of the enhancement of the electric field normalized to the incident field in the *x*–*y* plane at c) 640 nm, d) 749 nm, e) 813 nm, and f) 890 nm, respectively.

The dielectric permittivity of Au and of the SiO_2_ spacer used in the simulations is described by the Lorentz–Drude model^[^ ^42^ ^]^ and the Kischkat model,^[^ ^43^ ^]^ respectively. The comparison between the experimental curve (**Figure** **2**a) and the numerical one (Figure 2b) shows a good agreement. The slight blue shift of the resonances in the experimental spectra could be due to fabrication imperfections and to the differences in the refractive indices considered in the model.

The spatial distribution in the *x*–*y* plane of the near field at the different resonances, corresponding to minima in the reflection spectrum, was carefully investigated. The electric field cross‐sectional maps (*zy*‐plane) of the different modes are reported in the SI. The normalized electric field distribution at 640 nm (Figure 2c) reveals a significantly higher electrical field intensity near the edges of the nanodisks compared to the surrounding areas. This confirms the existence of high electric field regions, which are a consequence of enhanced electron oscillations caused by the LSPR and the array effect.^[^ ^44^ ^]^

Except for the first mode, the remaining three modes are a combination of LSPR and SPP. In this composite structure, SPPs can be effectively excited. The incident energy initially couples to the LSPs through the individual nanodisks, which then interact with adjacent nanodisks both directly and through the SPP surface wave. Additionally, the array of nanodisks acts as a two‐dimensional grating structure, providing extra momentum that facilitates the transfer of incident energy to the SPPs.^[^ ^45^, ^46^, ^47^ ^]^ This coupling results in a very low reflectivity at the resonant wavelength in the reflection spectrum. In particular, the 749 nm mode shows SPP mode behavior (Figure 2d), while the 813 nm mode shows a field enhancement related equally to a LSPR and a SPP mode (Figure 2e). It is possible to find the same maximum value |*E*|/*E*_0_ of 39.3 in proximity of the nanodisks and among them. A field configuration that is much more spatially distributed, and therefore with fewer hot‐spots on the surface, is instead found at the wavelength of 890 nm. This latter mode is characterized by a significantly lower Q‐factor,^[^ ^48^ ^]^ calculated as the ratio between the characteristic wavelength of the mode *λ*_res_ and its full width at half maximum (FWHM), than the other modes. The Q‐factor calculated from the numerical data is: 64 for the mode at 640 nm, 250 for the mode at 749 nm, 203 for the mode at 813 nm, and 22 for the mode at 890 nm. The experimental Q‐factor results to be equal to 15 for the mode at 657 nm, 62 for the mode at 747 nm, 158 for the mode at 788 nm, and 38 for the mode at 830 nm. From a comparison between the numerical and experimental Q‐factor results, it can be seen that the mode related to the LSPR is the most sensitive to fabrication defects. These includes the polydispersivity in the heights and diameters of the disks with a consequent enlargement of the curve and a worsening of the Q‐factor value.

### 2.2 Scanning Near‐Field Optical Microscopy (SNOM) Analysis

**Figure 3:**
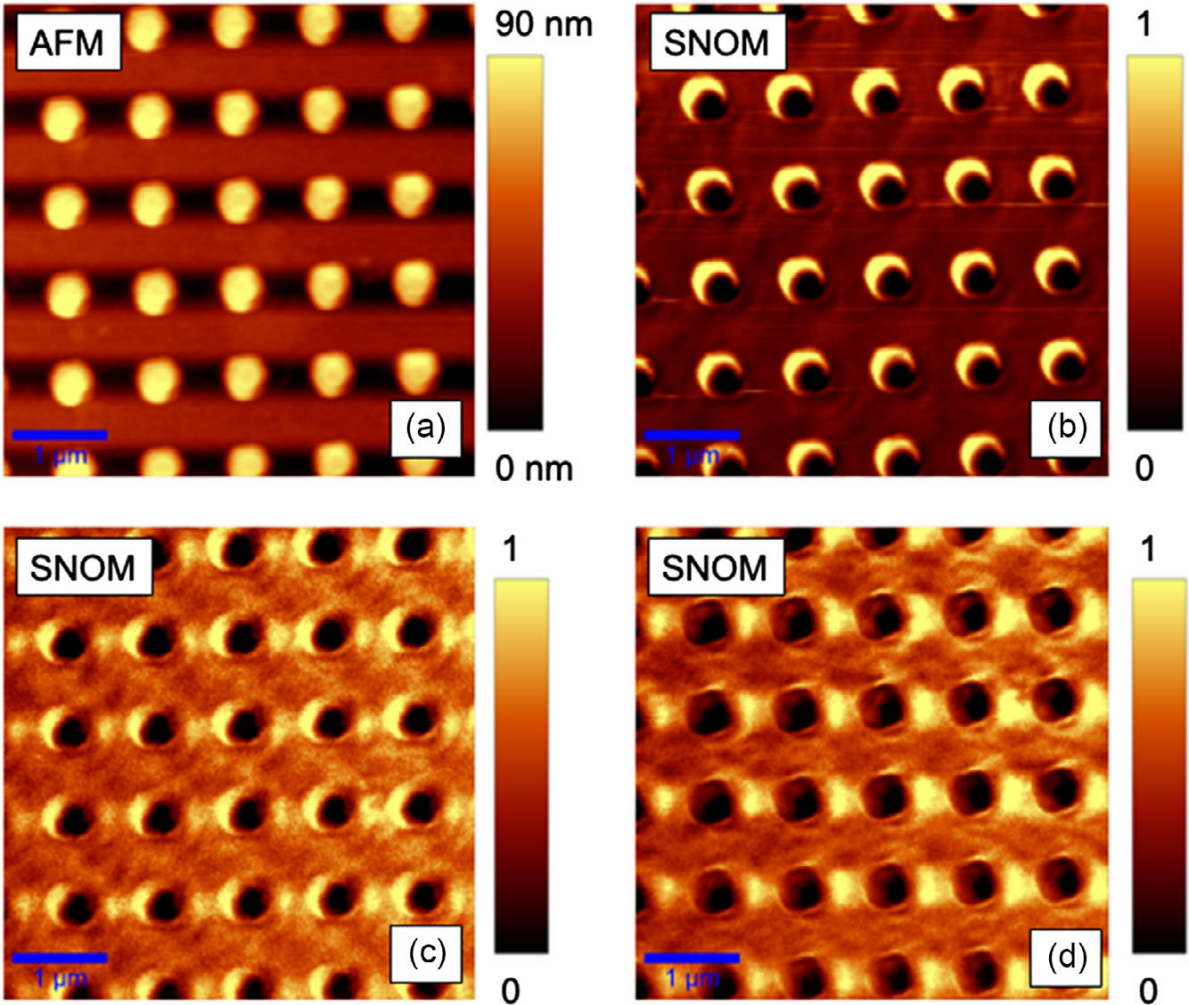
Experimental near‐field response of the composite structure. a) Topography and the corresponding SNOM images acquired at normal incidence at b) 532 nm, c) 750 nm, and d) 975 nm, respectively.

To further characterize the optical response and validate the structural integrity of the Au nanodisk array, we utilized SNOM in reflectance mode. This technique allows for high‐resolution imaging beyond the diffraction limit, providing detailed insights into the local optical properties and interactions within the nanostructured array. The topographic atomic force microscopy (AFM) and SNOM images acquired in reflectance mode are presented in **Figure** **3**. The AFM image (Figure 3a) reveals several key features of the nanodisk array: each nanodisk exhibits consistent dimensions and shape, with a height of 40 nm, as designed. The SNOM analysis is carried out at different excitation wavelenght. The first one, at *λ*_exc_ = 532 nm, reported in Figure 3b is characterized by an intense signal around the nanodisks and displays strong localized plasmonic resonances. These resonances manifest as bright hot‐spots in the SNOM images, corresponding to areas of enhanced electromagnetic fields. The bright spots are most intense at the edges of the nanodisks, which is consistent with the expected behavior of LSPRs. A different behavior is obtained at *λ*_exc_ = 750 nm, where in the regions between adjacent nanodisks, the SNOM images reveal interference patterns resulting from the coupling of localized and propagating plasmonic modes. At *λ*_exc_ = 795 nm, it is possible to see hot‐spots more marked between the structures than on the edges of the latter. The underlying 100 nm thick Au layer contributes to the overall reflectance signal, as seen in the consistent background intensity across Figure 3c,d. The presence of the 20 nm silica spacer layer is critical in modulating this interaction, providing a clear distinction between the reflectance signals from the nanodisks and the substrate.

### 2.3 Bulk RI Sensing

To evaluate the sensing performance of the designed platform, a series of experiments were conducted using a water‐based solution of glycerol at fixed concentrations of 1.0, 5.0, and 10.0 wt%. A volume of 100 μL of each glycerol solution was sequentially injected into the microfluidic chamber, with intermediate washing steps using deionized water (DI) to ensure baseline re‐establishment. The reflected signal was recorded at a fixed angle of 60° and p‐polarization to monitor the spectral shifts induced by changes in the RI surrounding the Au nanodisks.

**Figure 4:**
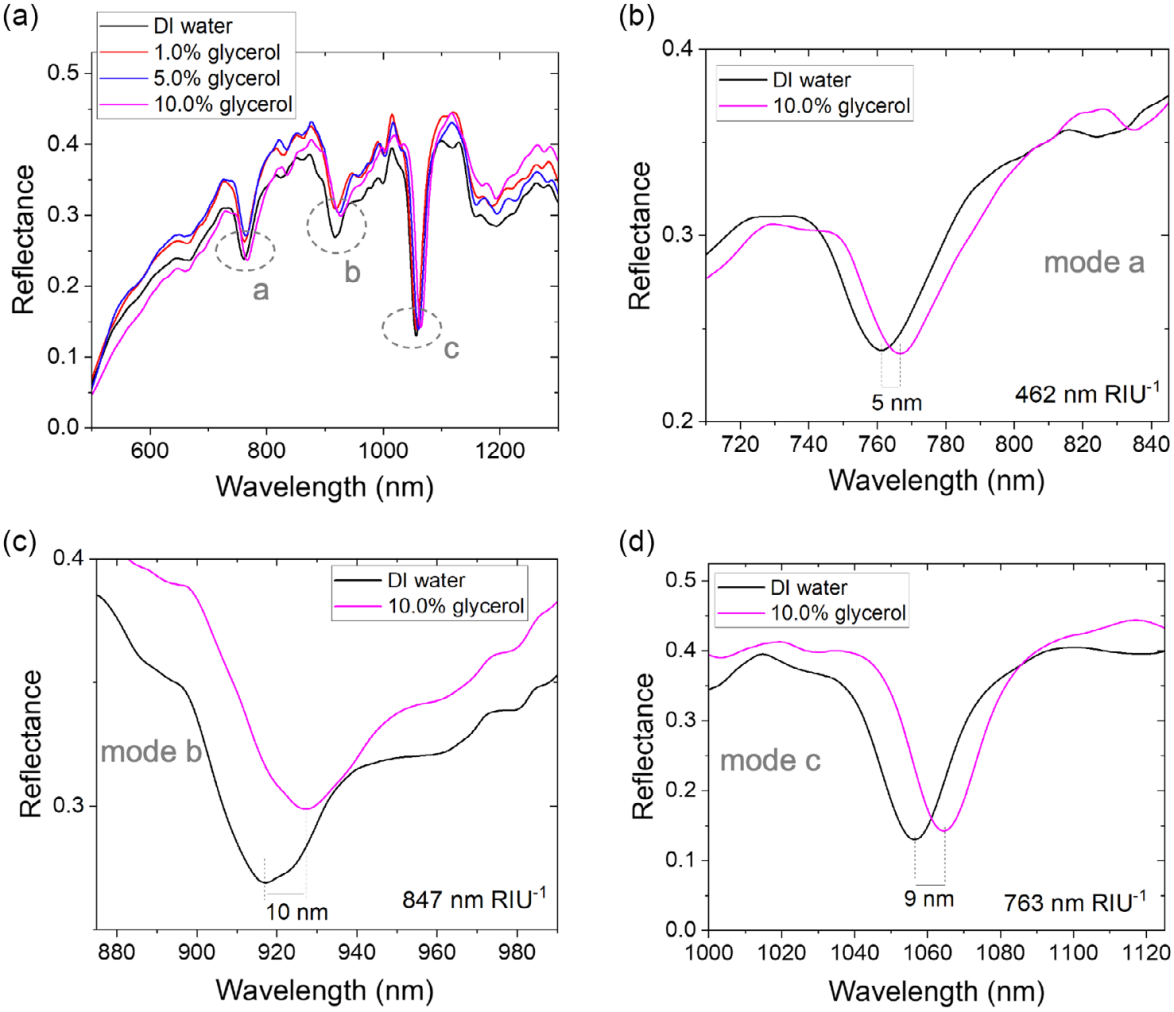
Bulk RI sensitivity. a) Reflected signal spectra showing the red‐shift as the RI increases from ultra‐pure water (1.3330) to 1.0% glycerol (1.3342), 5.0% glycerol (1.3388), and 10.0% glycerol (1.3448). b) Sensitivity of mode a at 760 nm, showing a value of 462 nm RIU^−1^. c) Sensitivity of mode b at 920 nm, showing a value of 847 nm RIU^−1^. d) Sensitivity of mode c at 1055 nm, showing a value of 763 nm RIU^−1^.

As shown in **Figure** **4**a, the introduction of glycerol solutions caused a significant red‐shift in the reflection spectrum. The RI of the medium increased from 1.3330 for DI water to 1.3342, 1.3388, and 1.3448 for 1.0%, 5.0%, and 10.0% glycerol solutions, respectively.^[^ ^49^ ^]^ This change in RI directly affected the plasmonic resonance modes of the Au nanodisks, resulting in measurable changes in the reflected signal. The sensitivity, *s* =  $\frac{\Delta \lambda }{\Delta n}$, expressed in nm RIU^−1^, where Δ*λ* is a change of resonant wavelength in response to a bulk RI change (Δ*n*), has been calculated. In particular, the sensitivity of the platform was evaluated based on the red‐shift observed for each mode. Specifically, the mode a at 760 nm demonstrated a sensitivity of 426 nm RIU^−1^ (Figure 4b), the mode b at 920 nm exhibited a sensitivity of 847 nm RIU^−1^ (Figure 4c), and the mode c at 1055 nm showed a sensitivity of 763 nm RIU^−1^ (Figure 4d). The metasurface‐based sensor demonstrated high sensitivity, with mode b at 920 nm exhibiting the highest sensitivity of 847 nm RIU^−1^. The sensitivity of the detection platform was evaluated against values reported in the literature for metasurface‐based sensors. Our sensor platform demonstrated a sensitivity of 847 nm RIU^−1^, surpassing other plasmonic biosensors based on LSPR (100–658 nm RIU^−1^),^[^ ^28^, ^31^, ^32^, ^33^, ^34^ ^]^ dielectric sensors (263–326 nm RIU^−1^),^[^ ^35^, ^36^, ^37^, ^38^ ^]^ and hybrid metal–dielectric nanoantennas (245 nm RIU^−1^),^[^ ^39^ ^]^ plasmonic 3D metasurfaces (761 nm RIU^−1^).^[^ ^40^ ^]^ This high sensitivity can be attributed to the optimized metasurface design, which combines LSPRs between the Au nanodisks and the SPPs of Au thin layer, enhancing the near‐field. The metasurface sensor's high sensitivity stems from the field enhancement in its 2D nanostructure, while the 2D design also offers fabrication advantages over 3D metasurfaces. This means it can effectively detect low molecular weight analytes without additional components like a prism, which is often used to enhance sensitivity. Specifically, the platform proposed in this article exhibited a sensitivity of 847 nm RIU^−1^, which not only exceeds the values reported in other works but also places the sensor as highly competitive within the field of metasurface‐based sensors. This sensitivity value indicates the platform's capability to detect minute changes in the RI of the surrounding medium, even without any surface functionalization. Additionally, the sensing performances were assessed in terms of another key parameter, the figure of merit (FOM), defined as the sensitivity divided by the FWHM of the resonance peak, calculated for each mode. The FOM values were 20, 37, and 36 RIU^−1^ for modes a, b, and c, respectively. These FOM values reflect the efficiency and accuracy of the detection platform in distinguishing between different refractive indices, further confirming its high performance for bulk glycerol sensing applications. Overall, the bulk glycerol sensing test demonstrated that the detection platform exhibits high sensitivity and a significant red‐shift response to changes in the RI. After these experiments, the next step was to evaluate a specific sensing performance for biotin recognition following the surface bio‐functionalization with streptavidin.

### 2.4 Low Molecular Weight Analyte Sensing

**Figure 5:**
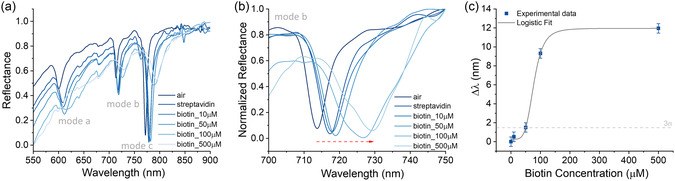
a) Reflectance spectra of Au nanodisk metasurface before and after functionalization with streptavidin, and at increasing concentrations of biotin; b) analysis of the reflectance mode b; and c) wavelengths shift of mode b as a function of biotin concentration, the gray line represents the logistic curve used to calculate the LOD.

The functionalization of the Au nanodisk metasurface with streptavidin was performed, as explained in the Experimental Section, to enable specific recognition of a low molecular weight analyte, the biotin (MW = 244 Da). The experiments involved exposing the metasurface to increasing concentrations of biotin ranging from 10 to 500 μM. Reflectance spectra were recorded at a fixed angle of 60° and with p‐polarized light, 60 min after deposition of each biotin solution. The deposition is carried out with a standard drop‐casting technique,^[^ ^50^ ^]^ where a 20 μL droplet of the prepared biotin solution in PBS is deposited onto the bio‐functionalized metasurface at room temperature and left air‐dry. After droplet evaporation, the unbound analyte is removed by a buffer washing step. After each biotin concentration, a control reflectance spectra were recorded to check that the signal returned back to that of streptavidin alone. Each streptavidin molecule present on the surface is expected to bind four molecules of biotin, with an affinity extremely high, with a dissociation constant (*K*_d_) in the range of 10^−14^–10^−15^ m.^[^ ^51^ ^]^ The initial preliminary measurements involved recording the reflectance spectrum of the metasurfaces after functionalization to verify the presence of streptavidin. These measurements were taken in four different regions to assess the uniformity of the biofunctionalization. Compared to the spectrum recorded in air, the presence of streptavidin on the Au nanostructures resulted in a noticeable red‐shift (see **Figure** **5**a). Following this, the spectra with biotin were recorded at increasing concentrations to observe the binding interaction. The results revealed a clear and consistent red‐shift in the reflectance spectra corresponding to the binding of increasing amounts of biotin to the functionalized Au nanodisks (see Figure 5a). This red‐shift is indicative of changes in the local RI around the nanodisks due to the binding of biotin molecules. Such spectral shifts are commonly observed in plasmonic sensing applications where the biomolecular binding alters the local dielectric environment of the plasmonic nanostructures.

To quantify the performance of our platform, the experimental data were fitted with a logistic function described by the equation:

(1) $$\Delta \lambda =\frac{\left(\Delta \lambda_{m}-\Delta \lambda_{M}\right)}{1+{\left(\frac{\left[B\right]}{{\left[B\right]}_{50}}\right)}^{s}}+\Delta \lambda_{m}$$

where:

Δ*λ* is the change in the plasmonic peak wavelength, representing the response signal of the LSPR sensor to the presence of the analyte.

Δ*λ*_M_ is the maximum signal response that can be achieved on the calibration curve when the analyte concentration is sufficiently high.

Δ*λ*_m_ is the minimum signal response observed on the calibration curve, typically corresponding to the baseline or to the response in the absence of the analyte.

[*B*] is the concentration of biotin in the sample being tested.

*S* is the slope‐like parameter that influences the steepness of the curve, affecting how quickly the response signal changes with the concentration of the analyte.

[*B*]_50_ is the concentration of biotin that produces a response signal that is halfway between Δ*λ*_m_ and Δ*λ*_M_, effectively the concentration at which a 50% signal response is observed.

The limit of detection (LOD), often known as sensor resolution, was calculated from the logistic equation considering three times the standard deviation of the baseline noise relative to the bulk sensitivity,^[^ ^52^ ^]^ (refer to Figure 5c). The LOD extracted for the mode b (see Figure 5b) is 50 μM, that corresponds to 12.2 μg mL^−1^. This demonstrates the sensor's high sensitivity in detecting low concentrations of small compound like biotin, for which conventional SPR sensor are not sensitive enough. The most common SPR methods typically require higher concentrations, often exceeding 100 μM, due to biotin's small size.^[^ ^53^ ^]^

In addition, the sensor shows a strong response in the concentration range of 50–150 μM, with signal saturation observed at higher concentrations. This dynamic range is relevant for certain applications, such as enzyme activity assays, drug pharmacokinetics, and detection of high‐abundance biomolecules in biological samples.^[^ ^54^, ^55^, ^56^, ^57^, ^58^, ^59^ ^]^

These findings demonstrate the effectiveness of the streptavidin‐functionalized Au nanodisk metasurface for specific and sensitive detection of low molecular weight analyte across a wide range of concentrations, showcasing its potential utility in biosensing applications.

## 3 Conclusion

In this study, we presented a novel sensing platform based on the coupling of localized and propagating plasmonic modes for the detection of molecules characterized by low molecular weight. Our design features a periodic array of Au nanodisks on a silica layer, supported by a gold substrate, optimized to enhance the plasmonic interactions and sensitivity. The Au nanodisks, with a diameter of 150 nm, height of 40 nm, and periodic spacing of 780 nm, were fabricated on a 20 nm thick silica layer atop a 100 nm Au thin layer. This configuration was meticulously designed to maximize the coupling between LSPRs in the nanodisks and propagating SPPs in the supporting layers. Using FEM analysis, we simulated the reflectance response of the nanodisk array over a wavelength range of 580–1100 nm. The results indicated distinct resonant peaks, corresponding to the plasmonic modes, validating our design approach and highlighting the platform's capability to enhance optical signals significantly. The SNOM analysis in reflectance mode provided high‐resolution images of the nanodisk array, revealing well‐defined plasmonic hotspots and interference patterns. These images confirmed the high uniformity and quality of the fabricated array and demonstrated the strong localized and propagating plasmonic interactions predicted by our simulations. To evaluate the bulk RI sensing performance, we tested the platform with glycerol solutions at different concentrations and achieved a sensitivity of 847 nm RIU^−1^. This high sensitivity underscores the platform's capability for detecting minute changes in the local RI, a crucial factor for effective sensing applications. The plasmonic coupling observed in our sensing platform offers a significant advantage in detecting low molecular weight molecules, where traditional sensing techniques often fall short. Specifically, our platform achieved a LOD of 50 μM (12.2 μg mL^−1^) for biotin, highlighting its sensitivity and potential for detecting low‐concentration analytes. The enhanced sensitivity and resolution provided by this platform open up new possibilities for applications in biosensing, environmental monitoring, and chemical detection. Finally, as the metasurface fabrication procedure is simple and compatible with microchips and microfluidic technologies, the metasurface‐based biosensor can be integrated into complex systems to detect different low molecular analytes. This potential integration opens up new possibilities for the development of portable, high‐performance biosensors that can be easily incorporated into various biomedical applications. Looking ahead, there are several promising directions to further enhance the sensor's sensitivity and lower the LOD. One approach involves fine‐tuning the size, shape, and arrangement of the nanodisks to optimize plasmonic coupling, thereby amplifying the sensor's interaction with target molecules. Additionally, incorporating specialized surface chemistry or bioreceptors with higher affinity for specific analytes could significantly improve biomolecule binding at lower concentrations. Finally, integrating advanced signal processing algorithms to detect subtle changes in RI could further push the detection limits. These strategies offer exciting opportunities to expand the sensor's applicability across a broader range of biomolecules and concentrations, paving the way for its use in more diverse clinical and environmental contexts.

## 4 Experimental Section

4.1

4.1.1

##### Device Fabrication and Morphological Characterization

Indium tin oxide‐coated glass surfaces with a resistivity of 8–12 ω sq^−1^ were purchased from Sigma Aldrich and submitted, as received, to SiO_2_ and Au thin layer deposition carried out by e‐beam evaporation in an ORION6 by AJA International. Both depositions were carried out in a vacuum of 2 × 10^−7^ mTorr at a rate of 2 Å s^−1^ controlling the layer thickness with a 6 MHz quartz microbalance. Arrays of plasmonic nanostructures were subsequently obtained by combining e‐beam lithography with metal evaporation and lift‐off. For e‐beam lithography, we used a positive two‐layer procedure to achieve an undercut structure profile to improve the lift‐off. Both resists are from microchemicals and are spin‐coated at 1500 rpm with an acceleration of 4950 rpm s^−1^ for one minute. After each spin coating step, we left the samples on a hot plate at 180 °C to allow solvent evaporation. This allowed obtaining a 500 nm thick EL6 (MMA (8.5) MMA) layer followed by an equal thickness layer of poly(methyl methacrylate) (PMMA) 950 k A2. Exposure was carried out in an e‐beam lithography system by Raith (150 TW) working at an operating voltage of 30KV. The samples were then developed for 30 s in metilisobutilchetone followed by another 30 s in isopropyl alcohol followed by evaporation of 40 nm of Au as described before. Finally, Au disk nanostructures with a 40 nm thickness were defined by lift‐off achieved by immersing the samples in an acetone bath. The latter step relies on the solubility of PMMA in acetone, allowing Au to be removed from the nonexposed pattern. Morphological characterization of the fabricated nanostructures array was performed using SEM (Raith 150).

##### Near‐Field Characterization: SNOM Analysis

The near‐field characterization was performed with a SNOM, Alpha300 by WITec, operating in the SNOM–AFM combination mode. The reflected signal from the plasmonic platform has been acquired under the same experimental conditions (incident power and polarization of excitation laser source, *λ*_exc_ = 532, 750, and 795 nm). The laser beam is focused on the samples through an Al‐coated aperture SNOM tip characterized by an aperture of length 60 nm, while reflected light is detected from the side by a photo‐multiplier tube.

##### Microfluidic Chamber Fabrication and Integration with the Substrate

The microfluidic chip is made up of a 1.5 mm thick polydimethylsiloxane frame into which, through the use of two needles connected to two syringes, it is possible to inject a volume of 100 μL of fluid. The upper part of the sensing platform is closed through a cover slip characterized by a thickness of 150 μm. To obtain the bond between the polymer and the glass surfaces, a plasma treatment was carried out.

##### Far‐Field Characterization: Ellipsometric Analysis

Variable‐angle high‐resolution spectroscopic ellipsometry (J. A. Woollam, V‐VASE) was used to measure the reflectivity spectra as a function of excitation wavelengths. The incident light was directed onto the sample at varying angles between 40° and 70° in increments of 10°. The reflected light was analyzed to determine the changes in the amplitude and phase of the polarization components. These measurements were performed across a wavelength range of 500–1000 nm, providing comprehensive data for evaluating the optical properties of the materials. This methodology, along with the authors’ previous work in biosensing and materials preparation, establishes a robust framework for analyzing and optimizing the performance of the sensing platform.^[^ ^60^, ^61^, ^62^, ^63^, ^64^, ^65^ ^]^

##### Chemicals

11‐Mercaptoundecanoic acid (MUA), Streptavidin (SA), Biotin, N‐(3‐dimethylaminopropyl)‐N‐ethylcarbodiimide hydrochloride (EDC), and N‐hydroxysuccinimide (NHS) were purchased from Sigma‐Aldrich (Saint Louis, MO, USA). Ultrapure water (Milli‐Q, 18 M ω ⋅ cm) was used for the preparation of the aqueous solutions and for all rinses. Dulbecco's phosphate‐buffered saline (DPBS, pH 7.4), ethanol, and dimethyl sulfoxide (DMSO) were purchased from Sigma‐Aldrich (St. Louis, MO, USA).

##### Biosample Preparation

A stock solution of SA was prepared in DPBS at a concentration of 1 mg mL^−1^. For Biotin, the stock solution was obtained by dissolving the powder in DMSO at a concentration of 5 mg mL^−1^ and then diluting it in DPBS to the final concentration.

One of the most common procedures to functionalize the surface of metal‐based substrates is the formation of self‐assembled monolayer (SAM) of molecules with appropriate terminal functional groups.^[^ ^66^, ^67^ ^]^ Due to the strong interaction between sulfhydryl group (‐SH) and noble metals, thiolate compounds are often used to start biofunctionalization processes, providing SAMs. Therefore, we used MUA to form on gold surfaces an alkanethiol monolayer with carboxyl‐terminal groups to subsequently anchor—through a covalent bonding—the SA protein. In particular, the carboxyl‐terminal groups were activated through EDC/NHS chemistry to react with the amino‐terminal group of the protein, with consequent formation of amide bonds.^[^ ^67^ ^]^ Briefly, the metastructure was immersed into an ethanolic solution 1 × 10^−2^ m of MUA for 24 h at room temperature. Then, it was rinsed with ethanol and immersed in a water solution. Under stirring, 1 mL of EDC and 1 mL of NHS dissolved in water were added to reach a final concentration of both of 4·10^−3^ m. After 1 h, the substrate was rinsed with water and immersed in a PBS solution 8.3·10^−8^ m of SA for 24 h under stirring at room temperature. Finally, it was rinsed several times with PBS—to remove unconjugated protein—and stored immersed in PBS.

##### Statistical Analysis

All data were analyzed and processed using Origin Software. In any given experiment, three independent measurements were executed, and all reported measurements were repeated three times to ensure reproducibility. Representative spectra are shown in the figures, and the data points and parameters are presented as mean ± SD (error bars).

## Conflict of Interest

The authors declare no conflict of interest.

## Author Contributions

**Alexa Guglielmelli**: Conceptualization (equal); Investigation (equal); Methodology (lead); Writing—original draft (equal). **Rossella Zaffino**: Data curation (lead); Investigation (lead); Methodology (lead); Writing—original draft (lead). **Giovanna Palermo**: Conceptualization (lead); Investigation (lead); Validation (lead); Writing—original draft (lead). **Liliana Valente**: Investigation (supporting); Methodology (supporting). **Dante Maria Aceti**: Investigation (supporting); Methodology (supporting). **Loredana Ricciardi**: Methodology (lead). **Arántzazu González‐Campo**: Supervision (lead); Writing—review & editing (lead). **Raphael Pfattner**: Supervision (supporting); Validation (supporting); Writing—review & editing (supporting). **Núria Aliaga‐Alcalde**: Supervision (lead); Writing—review & editing (lead). **Giuseppe Strangi**: Project administration (lead); Supervision (lead); Writing—review & editing (lead).

## Supporting information

Supplementary Material

## Data Availability

The data that support the findings of this study are available from the corresponding author upon reasonable request.
