# Supplementary material for: Plasmonic Coupling for High‐Sensitivity Detection of Low Molecular Weight Molecules
Source: Small Sci. 2024 Oct 9;5(1):2400382. doi: 10.1002/smsc.202400382 (PMC11935176; doi:10.1002/smsc.202400382)
Supplement: Supplementary file 1 — Supplementary Material [file SMSC-5-2400382-s001.pdf]

Plasmonic Coupling for High-Sensitivity Detection of Low Molecular Weight Molecules

Alexa Guglielmelli Rossella Zaffino Giovanna Palermo* Liliana Valente Dante Maria Aceti Loredana Ricciardi ArÅLantzazu GonzÅLalez-Campo Raphael Pfattner NÅLuria Aliaga-Alcalde Giuseppe Strangi**

A. Guglielmelli, G. Palermo, L. Valente, D. M. Aceti
Department of Physics, NLHT-Lab, University of Calabria and CNR-NANOTEC, Institute of Nanotechnology, 87036 Rende, Italy.
Email:giovanna.palermo@unical.it
L. Ricciardi
CNR-NANOTEC, Institute of Nanotechnology, 87036 Rende, Italy.
R. Zaffino, A. GonzÅLalez-Campo, R. Pfattner
Institut de Ciència de Materials de Barcelona (ICMAB-CSIC) Campus Universitari, 08193 Bellaterra, Spain.
Email: rzaffino@icmab.es
N. Aliaga-Alcalde Institut de Ciència de Materials de Barcelona (ICMAB-CSIC) Campus Universitari, 08193 Bellaterra, Spain. ICREA (InstituciÅLo Catalana de Recerca i Estudis Avan.cats) Passeig LluÅNıs Companys 23, 08010 Barcelona, Spain.
G. Strangi
Department of Physics, NLHT-Lab, University of Calabria and CNR-NANOTEC, Institute of Nanotechnology, 87036 Rende, Italy. Department of Physics, Case Western Reserve University, 2076 Adelbert Rd, Cleveland, Ohio, 44106, USA.
Email: gxs284@case.edu

Numerical EM Modeling.

To simulate the metasurface in Comsol Multiphysics, we chose a 3D geometry consisting of a parallelepiped with the height depending on the specific wavelength considered in the calculation.
The parallelepiped is constituted by 3 regions: starting from the bottom: i) the glass with a refractive index n_{glass} = 1.517; ii) the gold layer of 100 nm (Johnson and Christy refractive index), iii) the SiO_2 spacer of 20 nm, and iv) the surrounding medium of the nanodisks array constitute of air (n_{air}= 1.0). To create a wave diffusion environment, an input and an output port were used, to simulate the incidence from the -*z*-direction (top). To ensure the infinity conditions in the (x, y) plane, the Periodic Boundary Conditions (PBCs) were applied to the surfaces along the (x, y) directions of the blocks. To simulate the repeated single unit cell, the Floaquet periodicity was used. In addition to the PBCs, Perfect Matched Layers (PMLs) were introduced into the simulated system, to reproduce an absorbing boundary condition at the block extremes. The equations were solved by discretizing the problem, creating a dense mesh with control over the single components of the constructed geometry. In particular, for this study, an “extremely-fine” mesh was chosen - Figure S1.
polarization of the impinging radiation can be suitably selected by considering the following amplitude for the magnetic field: (0,1,0) for p-polarized light.

The Reflectance through the structure is calculated with the Scattering Matrix coefficient.

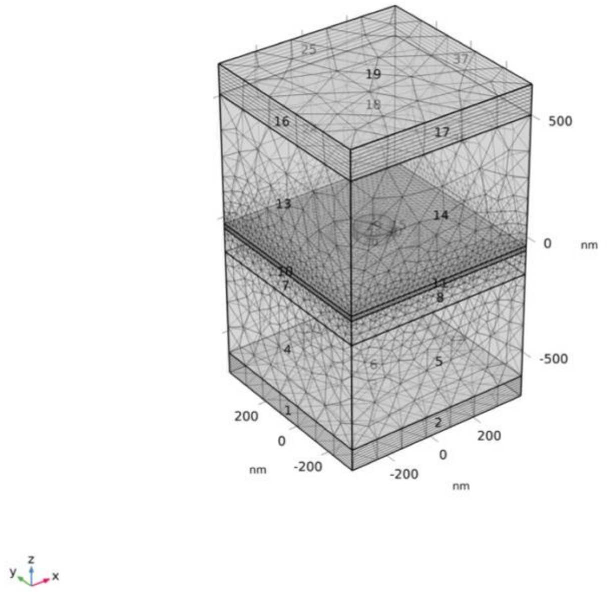

Figure S1: Unit cell of the Comsol Multiphysics model.

Electric field cross-sectional maps (zy-plane) of the resonance modes.

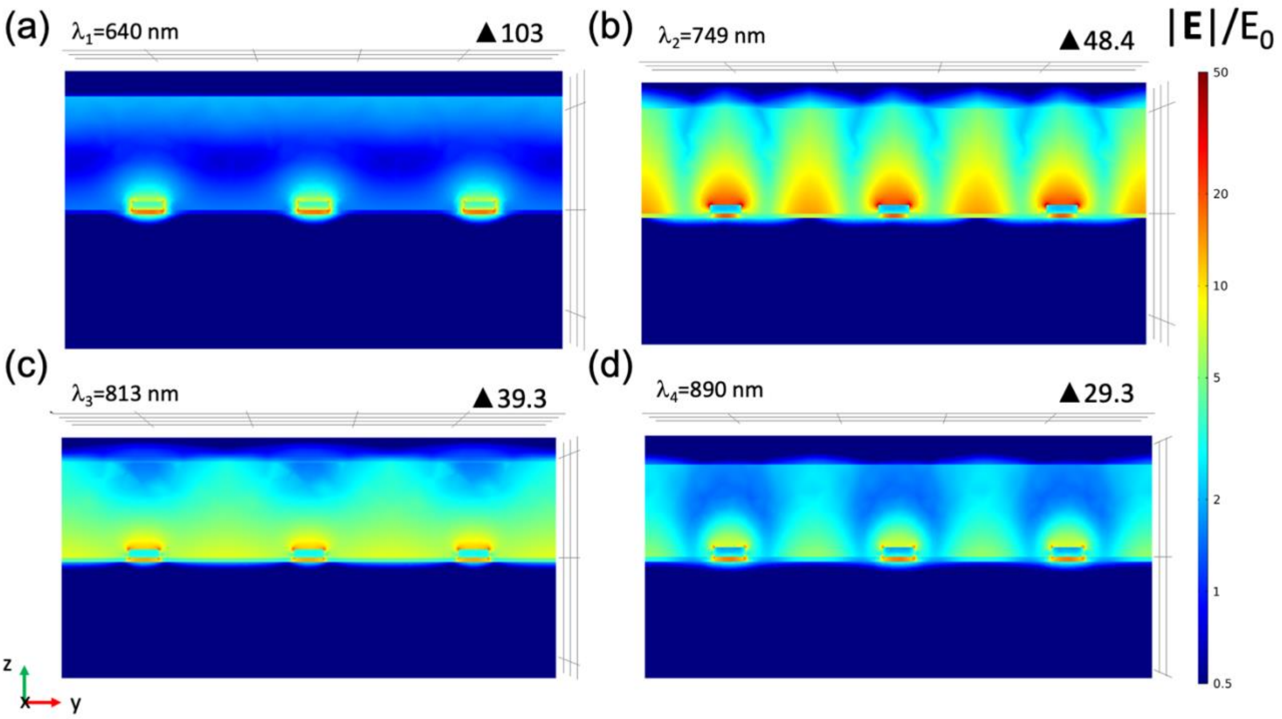

Figure S2: Electric field cross-sectional maps (zy-plane) of the resonance modes.
